# Crimean-Congo Hemorrhagic Fever Virus in High-Risk Population, Turkey

**DOI:** 10.3201/eid1503.080687

**Published:** 2009-03

**Authors:** Turabi Gunes, Aynur Engin, Omer Poyraz, Nazif Elaldi, Safak Kaya, Ilyas Dokmetas, Mehmet Bakir, Ziynet Cinar

**Affiliations:** Cumhuriyet University, Sivas, Turkey

**Keywords:** Crimean-Congo hemorrhagic fever, epicenter, seroprevalence, Turkey, dispatch

## Abstract

In the Tokat and Sivas provinces of Turkey, the overall Crimean-Congo hemorrhagic fever virus (CCHFV) seroprevalence was 12.8% among 782 members of a high-risk population. CCHFV seroprevalence was associated with history of tick bite or tick removal from animals, employment in animal husbandry or farming, and being >40 years of age.

Crimean-Congo hemorrhagic fever virus (CCHFV) infection was first defined in Turkey in 2003 from persons who became sick during a 2002 CCHFV outbreak (*1*,*2*). During 2002–2007, CCHFV was confirmed serologically, virologically, or by both types of testing, in ≈1,800 persons, mainly in the Tokat and Sivas provinces of Turkey (Figure 1) (*3*). This region was then considered an epicenter for CCHFV epidemics (*4*). This study determined the seroprevalence of CCHFV in a high-risk population living in that region after 4 epidemic seasons and assessed transmission routes of CCHFV infection.

**Figure 1:**
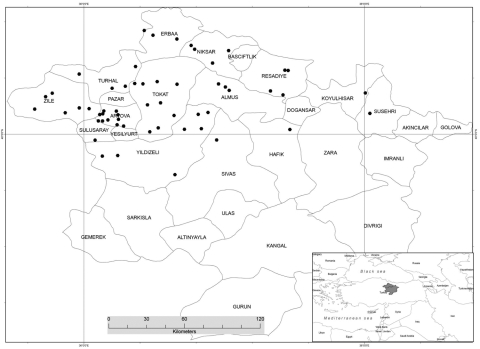
Districts of Tokat and Sivas provinces, Turkey, from which 782 persons at high risk for Crimean-Congo hemorrhagic fever virus (CCHFV) infection were sampled, 2006. Sample sites are indicated by black dots. (Map provided by Zati Vatansever and reproduced with permission.)

## The Study

In June and September 2006, persons living in 56 villages of the 14 districts of Tokat and Sivas provinces (Figure 1) who had a risk for CCHFV infection other than occupational risk (i.e., healthcare, slaughterhouse work, and veterinary care) were randomly selected for the study. Villages and districts were selected based on residences of patients who were diagnosed with CCHFV infection and treated at Cumhuriyet University Hospital, Sivas, Turkey, during the 2005 CCHFV outbreak. Men and women were included in the study, but children <7 years of age were excluded because of difficulties in drawing blood samples and obtaining parental consent.

Using EPI Info version 6 software (Centers for Disease Control and Prevention, Atlanta, GA, USA) and assuming a CCHFV seroprevalence of 10% in the study population with 99% confidence levels, we calculated error limits of ± 3% and a design effect of 1. The estimated sample size required was 664, but the target sample size of high-risk persons was increased to 782. Another 100 persons who were not at high risk for CCHFV infection, but who lived in urban areas in the high-risk region and agreed to provide blood samples, were also included in the study. The study protocol was approved by the Cumhuriyet University Hospital Human Ethics Committee.

The CCHFV Seroprevalence Study Team in Turkey included a physician and a nurse who went to the selected villages and approached the heads of the village and selected families. They explained the objectives of the study and asked for written informed consent from participants or parents of participating minors and then administered an interview-based questionnaire and collected a blood sample. The questionnaire considered the following variables: age; sex; history of tick bite, tick removal from animals, animal abortion, and animal slaughtering activity; close contact with a CCHFV patient or an animal; and occupation. Blood samples (10 mL each) were collected and later tested for antibodies to CCHFV by using immunoglobulin G (IgG) ELISA kits (Vector-Best; Kolsovo, Novosibirsk, Russia). SPSS version 10.0 (SPSS, Chicago, IL, USA) for Windows software was used for statistical analysis. Chi-square and Fisher exact tests were used to compare categorical variables. Statistical significance was defined as a 2-tailed p value <0.05. Univariate analysis was used to identify the risk factors for seropositivity of CCHFV in the 782 participants.

**Figure 2:**
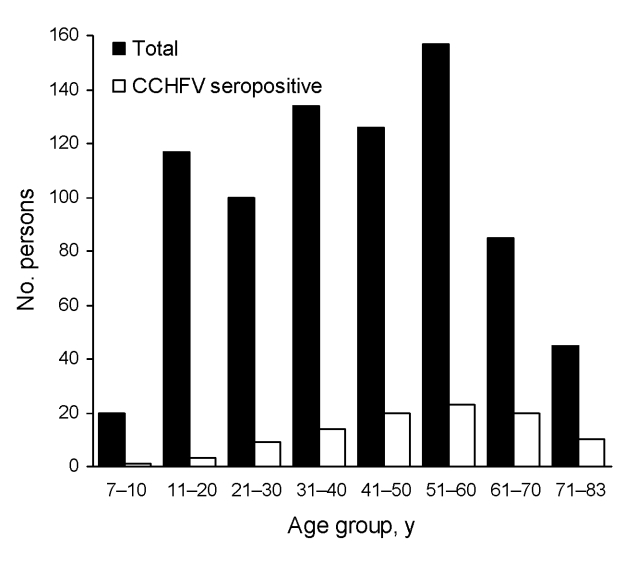
Distribution of seroprevalence of immunoglobulin G against Crimean-Congo hemorrhagic fever virus by age groups for 782 high-risk persons living in rural areas of Tokat and Sivas provinces, Turkey, 2006.

Of the 782 high-risk persons, 100 were positive for IgG against CCHFV (seroprevalence 12.8%). The sex ratio was ≈1:1 (390 females, 392 males). Forty-seven (12.1%) of 390 female participants and 53 (13.5%) of 392 male participants were seropositive for CCHFV (p>0.05). Mean age was 41.5 years. Of the 100 serum samples collected in the urban population, only 2 (males 44 and 56 years of age) were seropositive. The CCHFV seroprevalence in the 782 persons at high risk increased significantly with age (p<0.001). The highest proportion (23.5%) of seropositivity was found in persons 61–70 years of age (p<0.001) (Table 1). Figure 2 shows distribution of the CCHFV seroprevalence in high-risk persons by age groups. The only variables significantly associated with presence of antibody against CCHFV were history of tick bite (p = 0.002) or of tick removal from the animals (p = 0.03), employment in animal husbandry (p = 0.01) or farming (p = 0.02), and age >40 years (p<0.001) (Table 2).

**Table 1:** Demographics and seroprevalence of CCHFV in persons living in rural and urban areas of Tokat and Sivas provinces, Turkey, 2006*

Characteristic	Persons living in rural area (n = 782)	Persons living in urban area (n = 100)
Age, y		
Mean ± SD	41.5 ± 18.6	41.9 ± 18.4
Range	7–83	7–80
Gender, no. (%)		
Female	390 (49.8)	53 (53)
Male	392 (50.2)	47 (47)
Total seroprevalence, no. positive (%)	100 (12.8)	2 (2)
Seroprevalence by gender, no. positive/no. tested (%)†		
Female	47/390 (12.1)	0/53 (0)
Male	53/392 (13.5)	2/47 (4.3)
Seroprevalence by age, y, no. positive/no. tested (%)‡		
7–20	4/138 (2.9)	0/14 (0)
21–30	9/100 (9)	0/18 (0)
31–40	14/134 (10.5)	0/15 (0)
41–50	20/126 (15.90	1/18 (5.6)
51–60	23/157 (14.6)	1/17(5.9)
61–70	20/85 (23.5)	0/13 (0)
71–83	10/45 (22.2)	0/5 (0)

*CCHFV, Crimean-Congo hemorrhagic fever virus. †p value = 0.59 for persons living in rural area; for persons living in urban area, data are insufficient for statistical analysis. ‡p value <0.001 for persons living in rural area; for persons living in urban area, data are insufficient for statistical analysis.

**Table 2:** Demographic features and risk factors associated with CCHFV seroprevalence (univariate analysis) for persons living in rural areas of Tokat and Sivas provinces, Turkey, 2006*

Risk factor category	No. seropositive persons/total population (%)	p value
Age >40 y	73/410 (17.8)	<0.001
History of tick bite	78/483 (11.5)	0.002
Tick removal from the animals	69/450 (15.3)	0.03
Animal abortion	19/135 (14.1)	0.67
Slaughtering activity	25/151 (16.6)	0.18
Contact with CCHFV patient	14/89 (15.7)	0.44
Contact with an animal	97/734 (16.6)	0.26
Job		
Farmer	93/656 (14.2)	0.02
Animal husbandry	94/664 (14.2)	0.01
Milking	35/263 (13.3)	0.79
Student	1/38 (2.6)	0.11
Total no. seropositive persons	100/782 (12.8)	–

*CCHFV, Crimean-Congo hemorrhagic fever virus.

## Conclusions

Serologic evidence of CCHFV in Turkey was reported in the 1970s (*4*). In 2003, the CCHFV seroprevalence among 40 veterinarians in the Tokat region was 2.5% (*5*). Another seroprevalence study conducted in 2003 among healthcare workers providing care to CCHFV patients in Turkey detected no seropositive persons (*6*). The present survey indicates that the seroprevalence of CCHFV is higher in persons living in rural areas than in urban areas of the CCHFV epicenter in Turkey (12.8% vs 2.0%). However, because special markets for animal trading are located on the outskirts of large cities in Iran, CCHFV seroprevalence was found to be higher among persons living in urban areas than in persons living in rural areas of this country (*7*). Living in a rural area is a risk factor for exposure to the tick vector and for acquiring CCHFV infection (*8*,*9*). Expected seroprevalence of CCHFV among high-risk persons during epidemics has been found to be 10% (*3*); however, seroprevalence has been reported to be as low as 0.5% in nonepidemic situations (*10*). Other studies conducted in rural parts of Iran and Senegal during epidemics showed that the CCHFV seroprevalence was 13%, comparable to our findings (*9**,**11*).

In the present study, history of tick bite and history of tick removal from animals were found to be significantly associated with CCHFV seropositivity. The overall tick-bite frequency was 62% (483/782) among persons at high risk and has been reported among 40%–60% of CCHFV patients in Turkey (*4*). We also determined that the occupations of animal husbandry and farming were significantly associated with CCHFV seropositivity. Vector ticks are generally present on the ground and on animals, which explains the risk for CCHFV infection in persons who work in farming and animal husbandry. Personal protective measures such as regular examination of clothing and skin for ticks, tick removal, and use of repellents are important to prevent CCHFV infection (*12*).

We did not identify any association between seroprevalence and gender but found that CCHFV seropositivity increased with age. In these regions of Turkey, women contribute to farming and animal husbandry tasks and are exposed to ticks and livestock as often as men are. However, age >40 years was significantly associated with CCHFV seropositivity and reflects the age of workers in Turkish agricultural areas (*4*,*8*,*13*). Increased CCHFV seroprevalence with age may result from increased opportunities of contact with vector ticks (*14*).

Exposure to blood and tissues of viremic animals during slaughter is a source of infection (*12*,*14*). However, we did not identify any association between CCHFV seropositivity and contact with animals. This finding may result from a low number of viremic animals in our study region. It is known that domestic animals generally have low levels of viremia, which lasts a short time (*15*). However, in our study region, 79% of animals have been found to be seropositive against CCHFV (*4*).

In the study population, 89 (11.4%) persons had a history of close contact with a CCHFV-infected patient. Among these 89 persons, 14 (15.7%) were seropositive, but this transmission route for CCHFV was not statistically significant for our study population. However, protection against this potential transmission route is especially important for healthcare workers in hospitals that provide care to CCHFV case-patients (*12*).

This study indicated that tick exposure is the most statistically significant transmission route for CCHFV in a high-risk population in Turkey. Effective tick prevention aids such as tick repellents may help reduce the risk. On the other hand, the absence of CCHFV seropositivity in 87.2% of the population after 4 CCHFV outbreaks in Turkey may suggest that this population remains at risk for infection in the future. This knowledge may help public health authorities determine appropriate CCHFV intervention and prevention methods.
